# Disseminated Coccidioidomycosis to the Gallbladder

**DOI:** 10.1177/2324709620910636

**Published:** 2020-03-05

**Authors:** Kulraj Grewal, Tushar Bajaj, Greti Petersen, Augustine Munoz, Arman Froush, Arash Heidari

**Affiliations:** 1 UCLA—Kern Medical, Bakersfield, CA, USA

**Keywords:** coccidioidomycosis, gallbladder, fungus, endemic mycosis

## Abstract

Coccidioidomycosis is an infection caused by inhalation of arthroconidia produced by dimorphic fungi in the genus *Coccidioides*. Forty percent of patients will develop an influenza-like illness with symptoms suggestive of a mild and self-limited respiratory infection; however, 5% of these individuals will develop extrapulmonary disseminated disease. An immunocompromised patient presented with right upper quadrant pain, ultrasound with pericholecystic fluid, in which a percutaneous cholecystostomy contained biliary fluid that grew the fungus *Coccidioides immitis*. Patient was initiated on intravenous amphotericin therapy and was followed closely with postoperative bile drainage with eventual laparoscopic cholecystectomy. We present a very rare case of disseminated coccidioidomycosis to the gallbladder.

## Introduction

Coccidioidomycosis is endemic to the southwestern United States, northern Mexico, Central America, and South America.^1-3^ These locations have the most frequent exposure to disrupted soil that increases the risk of an infection by coccidioidomycosis.^4^ The infection is caused by the inhalation of *Coccidioides immitis* or *Coccidioides posadasii*, which are the 2 species of dimorphic fungal spores found in individuals based on their endemic site of infectivity.^5^ The infection is self-limited in majority of patients and generally resolves without requiring specific treatment. There are 4.7% of recorded cases, especially with immunodeficiency, that have dissemination outside the confines of the chest cavity.^6^ The pulmonary inhalation disseminates through the lymphatic system and is visually apparent with hilar adenopathy present on radiographic imaging. Extrapulmonary dissemination can spread to skin or subcutaneous tissue, meninges, skeleton, endocrine glands, eye, liver, kidneys, genital organs, prostate, and the peritoneal cavity.^7-16^

## Case Presentation

A 60-year-old Hispanic male with uncontrolled diabetes mellitus type 2 and history of disseminated coccidioidomycosis to right hand and ankle (1995) presented with acute onset of dyspnea associated with right upper quadrant pain lasting 2 weeks and progressively worsening fatigue. Initially patient presented with diabetic ketoacidosis and was treated appropriately in the intensive care unit. Initial plain chest roentgenogram demonstrated no evidence of infiltration, consolidation, pulmonary edema, or pleural effusion.

**Figure 1.**
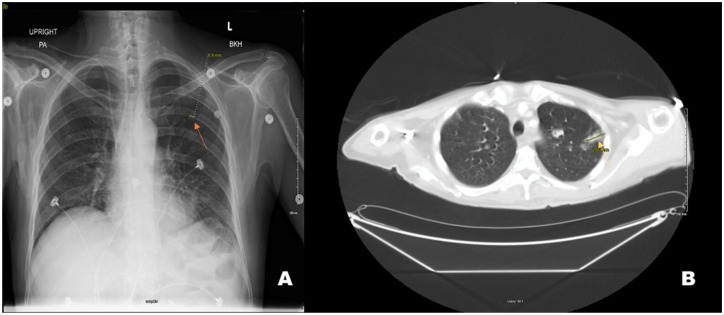
A posterior-anterior view of chest X-ray illustrating a lingular patchy alveolar density and a 6.9-mm left mid-lung pulmonary nodule (orange arrow) in “A.” An axial computed tomography of the chest demonstrates numerous pulmonary nodules, many of them with cavitation, the largest of which is pictured in “B” with a 2.8-cm left upper lobe pulmonary nodule (yellow arrow).

After stabilization and resolution of diabetic ketoacidosis, the patient was transferred to a medical ward in which he started to spike high-grade fevers and complained of new symptoms of hiccups with progressively worsening abdominal pain localized to the right upper quadrant. Physical examination of the lungs was clear to auscultation, and the abdomen was nontender to palpation. A repeat plain chest roentgenogram demonstrated a lingular patchy alveolar density with a 6.9-mm left pulmonary nodule on a posterior-anterior view (Figure 1A).

**Figure 2.**
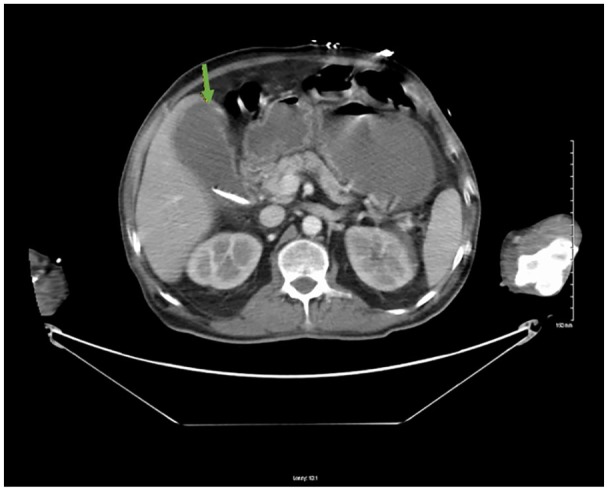
Computed tomography of the abdomen with contrast demonstrating mildly distended gallbladder containing stones with minimal wall thickening (green arrow).

The patient was initiated on broad-spectrum antibiotics; however, the patient had persistent fevers, a nonproductive cough, and hiccups. An abdominal X-ray was negative for any acute pathology. Due to the inconsistency of patient’s presentation with a normal physical examination, a computed tomography of the chest, abdomen, and pelvis with contrast was obtained and revealed numerous cavitary pulmonary nodules with one large pulmonary nodule in the left upper lobe measuring 2.8 cm (Figure 1B). Furthermore, the abdomen had a mildly distended gallbladder with gallstones, gallbladder wall thickening, however, there was no adenopathy or organomegaly (Figure 2).

**Figure 3.**
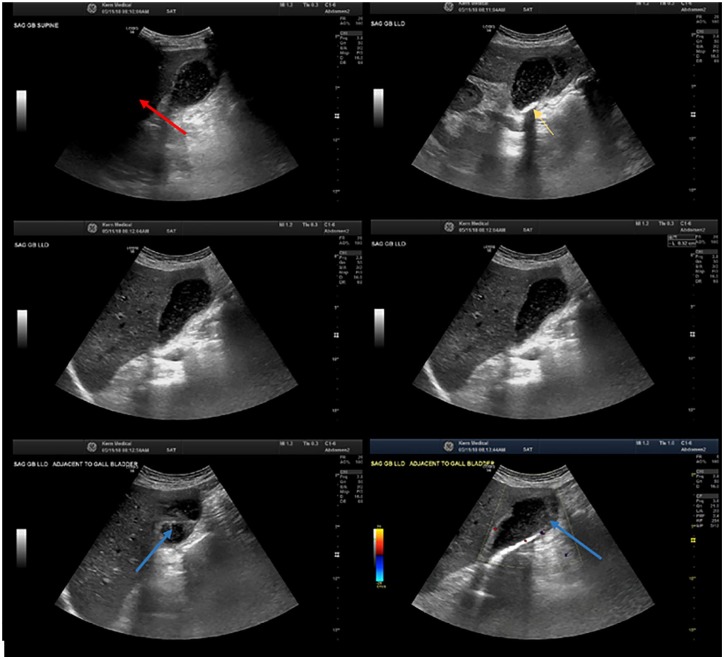
Right upper quadrant sonogram revealing biliary sludge (blue arrows), calcified stones (yellow arrow), and a sizable area of fluid collection adjacent to the gallbladder measuring 5.6 cm × 3.5 cm in size (red arrow).

**Figure 4.**
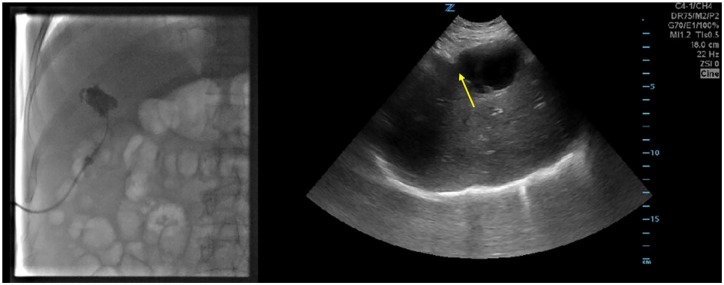
Fistulogram demonstrating sinus tract (yellow arrow) leading from the lumen of the gallbladder to the pericholecystic cavity.

A follow-up sonogram of the gallbladder contained biliary sludge, calcified stones, and a fluid collection adjacent to the gallbladder measuring 5.6 cm × 3.5 cm (Figure 3). The patient then underwent an ultrasound-guided percutaneous drainage of the pericholecystic fluid collection. A total of 80 mm of cloudy fluid was drained; however, there was a discordance between volume removed and appearance of fluid on the ultrasound. Furthermore, a fistulogram was performed that demonstrated a tract leading to the lumen of the gallbladder, opacification of the gallbladder lumen, with formation of a sinus tract from the gallbladder to the pericholecystic cavity (Figure 4).

**Figure 5.**
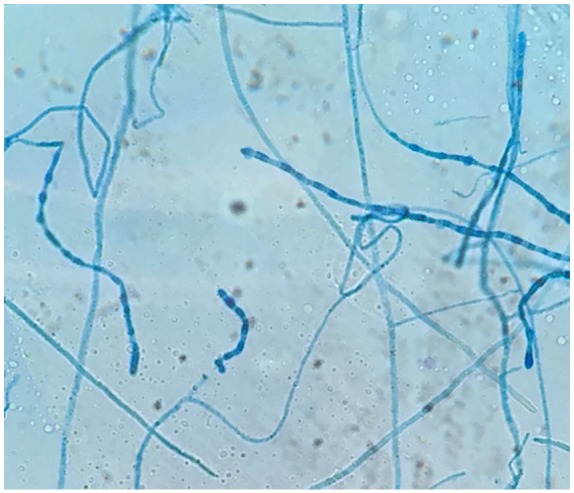
Gallbladder pericholecystic fluid fungal culture growing *Coccidioides immitis*.

The patient’s serum serology for coccidioidomycosis was positive with a titer of 1:16 (normal value <1:2). Cytology from the biliary fluid confirmed presence of fungal hyphae, with a final fungal culture revealing *C immitis* (Figure 5). Patient was initiated on intravenous amphotericin, and the patient remained afebrile for the duration of his hospital course. The patient’s other symptoms including the hiccups resolved as well. A cholecystostomy drain was left in place that actively drained turbid bilious fluid. The patient underwent laparoscopic cholecystectomy 4 months after discharge with removal of cholecystostomy drain. The patient is planned to continue lifelong amphotericin infusions 3 times weekly for his coccidioidomycosis infection due to the dissemination to the bones and gallbladder.

## Discussion

Approximately 20% of patients infected with coccidioidomycosis may initially present with fevers, night sweats, and weight loss; however, some may exhibit no radiographic findings where others will have patchy segmental opacities with regional lymphadenopathy.^17-19^ Similar to our patient with a left mid-lung pulmonary nodule, many of the lung nodules in coccidioidomycosis are found in the upper or middle lobes of patients with increased risk factors such as diabetes mellitus or smokers with chronic disease.^20^ The majority of patients have a self-limited clinical course without any medical intervention; however, patients who are immunocompromised with associated risk factors are more probable to have dissemination outside the confines of the chest cavity and persistently have ongoing chronic etiology.

One of the most common sites of dissemination includes cutaneous manifestations, which our patient previously had earlier in his life.^21,22^ Up to 50% of patients with disseminated disease demonstrate bone and joint involvement; furthermore, the infectious process can involve the axial skeleton with multiple bone lesions. The most common reported radiographic pattern is multiple punched-out lytic lesions with circumscribed margins in long and flat bones; however, bone destruction is another recognized pattern of osseous involvement with coccidioidomycosis in association with periosteal reaction and soft tissue disease.^23,24^ Often, the extent of dissemination relies on radiographic imaging, and there have been instances in which a highly suspicious lesion may be detected on one modality and not on another.^25^ Radionuclide scanning with technetium-99m methylene-diphosphonate or gallium-67 citrate can be performed to detect osseous involvement with demonstration of increased tracer activity that may be clinically occult.^18^

Since our patient had a history of disseminated coccidioidomycosis, the clinical presentation of new symptoms including right upper quadrant pain and persistent hiccups, it is important to consider fungal dissemination. Occurrence of peritoneal coccidioidomycosis has been described in literature and has been illustrated on computed tomography as diffuse parenchymal involvement or enlarged abdominal lymph nodes.^26,27^ To date, there has only been one other reported case of disseminated coccidioidomycosis to involve the hepatobiliary system with particular dissemination to the gallbladder as described by Sydorak et al in 2001.^5^

Ultrasound-guided percutaneous interventional procedures are minimally invasive and can be performed as adjunctive treatment with excellent long-term outcome and lower complication rates in comparison to open surgical procedures.^28-30^ This modality is often used in conjunction with a cholangiogram to aid in identification of ductal structures and should be further obtained to document anatomy.^31^ In our patient, a percutaneous cholecystostomy drain was placed and aided in relief of symptoms and expedited fungal culture diagnostic workup for cytology. A definitive diagnosis of coccidioidomycosis can be achieved by positive sputum culture, a positive reaction to coccidioidin skin test, or coccidioidal serology.^32,33^ The patient’s serum confirmed the diagnosis in our patient’s case with a titer of 1:16, and cytology confirmed fungal hyphae with final culture of *C immitis*. Disseminated disease has a poorer prognosis with high mortality and may even require lifelong antifungal treatment.^33^ The patient was initiated on amphotericin infusions 3 times per week and still continues to receive treatment as an outpatient.

The patient had a significant risk factor of uncontrolled diabetes mellitus, and in our case, the presentation of diabetic ketoacidosis created a temporary immunocompromised state of health that could have attributed to the dissemination of the fungal infection. The patient was previously a 12 pack-year smoker; however, he quit 10 years prior to the current presentation. However, the presence of a sinus tract connecting the gallbladder to a collection of fluid suggests a history of prior perforation with dissemination to the gallbladder in a more chronic etiology. This rare case report demonstrates the necessity of high clinical suspicion to include disseminated coccidioidomycosis in the differential in a patient with certain geographical location, risk factors, and prior history of infection with coccidioidomycosis.

## Conclusion

Most infections due to *C immitis* are self-limited and resolve over a period of weeks to months without any specific treatment. In rare cases, especially those associated with immunodeficiency or associated risk factors, the disease can be found outside the confines of the chest cavity. Therefore, a careful evaluation including a detailed history, physical examination, and radiographic modalities can aid in establishing the extent of disseminated coccidioidomycosis diagnosis. This particular case demonstrated an atypical manifestation of disseminated coccidioidomycosis to the gallbladder among other more commonly known sites of dissemination. This rare manifestation helps motivate further investigation into the pathophysiology and current treatment guidelines of coccidioidomycosis involved in dissemination to the hepatobiliary system.
